# Adverse Effects of Anabolic-Androgenic Steroids: A Literature Review

**DOI:** 10.3390/healthcare9010097

**Published:** 2021-01-19

**Authors:** Giuseppe Davide Albano, Francesco Amico, Giuseppe Cocimano, Aldo Liberto, Francesca Maglietta, Massimiliano Esposito, Giuseppe Li Rosi, Nunzio Di Nunno, Monica Salerno, Angelo Montana

**Affiliations:** 1 Legal Medicine, Department of Medical, Surgical and Advanced Technologies, “G.F. Ingrassia”, University of Catania, 95123 Catania, Italy; gdavidealbano@gmail.com (G.D.A.); francesco.amico@you.unipa.it (F.A.); giuseppe.cocimano@you.unipa.it (G.C.); aldoliberto@gmail.com (A.L.); massimiliano.esposito@you.unipa.it (M.E.); monica.salerno@unict.it (M.S.); 2 Department of Clinical and Experimental Medicine, University of Foggia, 71122 Foggia, Italy; francesca.maglietta@unifg.it; 3 Department of Law, Criminology, Magna Graecia University of Catanzaro, 88100 Catanzaro, Italy; lirosigiose@gmail.com; 4 Department of History, Society and Studies on Humanity, University of Salento, 73100 Lecce, Italy; nunzio.dinunno@unisalento.it

**Keywords:** AASs, anabolic androgenic steroids, organ damage, toxicity, injury, chronic administration

## Abstract

Anabolic-androgenic steroids (AASs) are a large group of molecules including endogenously produced androgens, such as testosterone, as well as synthetically manufactured derivatives. AAS use is widespread due to their ability to improve muscle growth for aesthetic purposes and athletes’ performance, minimizing androgenic effects. AAS use is very popular and 1–3% of US inhabitants have been estimated to be AAS users. However, AASs have side effects, involving all organs, tissues and body functions, especially long-term toxicity involving the cardiovascular system and the reproductive system, thereby, their abuse is considered a public health issue. The aim of the proposed review is to highlight the most recent evidence regarding the mechanisms of action of AASs and their unwanted effects on organs and lifestyle, as well as suggesting that AAS misuse and abuse lead to adverse effects in all body tissues and organs. Oxidative stress, apoptosis, and protein synthesis alteration are common mechanisms involved in AAS-related damage in the whole body. The cardiovascular system and the reproductive system are the most frequently involved apparatuses. Epidemiology as well as the molecular and pathological mechanisms involved in the neuropsychiatric side-effects of AAS abuse are still unclear, further research is needed in this field. In addition, diagnostically reliable tests for AAS abuse should be standardized. In this regard, to prevent the use of AASs, public health measures in all settings are crucial. These measures consist of improved knowledge among healthcare workers, proper doping screening tests, educational interventions, and updated legislation.

## 1. Introduction

Anabolic-androgenic steroids (AASs), commonly known as anabolic steroids, are a large group of molecules including endogenously produced androgens, such as testosterone, as well as synthetically manufactured derivatives [1]. Testosterone, Nandrolone Decanoate (ND), methandienone, and methenolol, are the most commonly abused androgens [2]. AAS use is widespread due to their ability to improve muscle growth for esthetic purposes and athletes’ performance, minimizing androgenic effects [3]. Indeed, androgens are able to increase the size of muscle fibers as well as muscle strength, and while their use was initially restricted to professional bodybuilders, nowadays it has become more popular among recreational athletes [4,5]. AAS anabolic properties have been widely used for therapeutic purposes. Indeed, AASs had a role in the treatment of chronic kidney disease and osteoporosis in postmenopausal women, as well as inoperable breast cancer, and for diseases characterized by a negative nitrogen balance [2]. However, use of AASs is forbidden by the World Anti-Doping Agency (WADA). However, AAS use is still very popular and 1–3% of US inhabitants have been estimated to be AAS users [6]. Moreover, in younger subjects’ higher estimates have been reported [7,8]. However, AASs have side effects involving all organs, tissues and body functions, especially long-term toxicity involving the cardiovascular system and the reproductive system, therefore, their abuse is considered a public health issue [9,10]. In this regard, an increased awareness is needed among the population and healthcare workers, both for diagnostic, therapeutic and prevention purposes. The aim of the proposed review is to highlight the state of the art regarding the mechanisms of action of AASs and the adverse effects related to AAS use/abuse. 

## 2. Phisiology of AASs

The anabolic androgenic effects are related to the androgen receptor (AR)-signaling action. Androgen receptors are widespread in human tissues and organs. There are three main action mechanisms: (i) direct binding to androgen receptor; (ii) via dihydrotestosterone (DHT) produced by the action of 5- a-reductase, and (iii) via estrogen receptors by means of estradiol produced by CYP19 aromatase. In particular, free testosterone is transported into target tissue cell cytoplasm; binding to the AR takes place either directly or after conversion to 5αdihydrotestosterone (DHT) by the cytoplasmic enzyme 5-alpha reductase. Into the cell nucleus, both free or bound, testosterone binds specific nucleotide sequences of the chromosomal DNA. The produced DNA activate the transcription of specific responsive genes, with significant influence on protein synthesis [11,12,13]. After dimerization the complex binds to specific promoter areas of target genes called androgen response elements (AREs), influencing the transcription process [14]. Furthermore, non-genomic pathways, by interfering with the G-protein coupled receptor, a transmembrane receptor located inside the cell, can lead to rapid steroid hormone activation [6,15]. In this regard, sex steroids might influence thyroid function as a consequence of the expression of androgen receptors in this tissue, leading to thyrocyte proliferation in culture independently from TSH [16]. The same mechanism has been described in other tissues [17].

## 3. Pathophysiology of AASs

**Figure 1:**
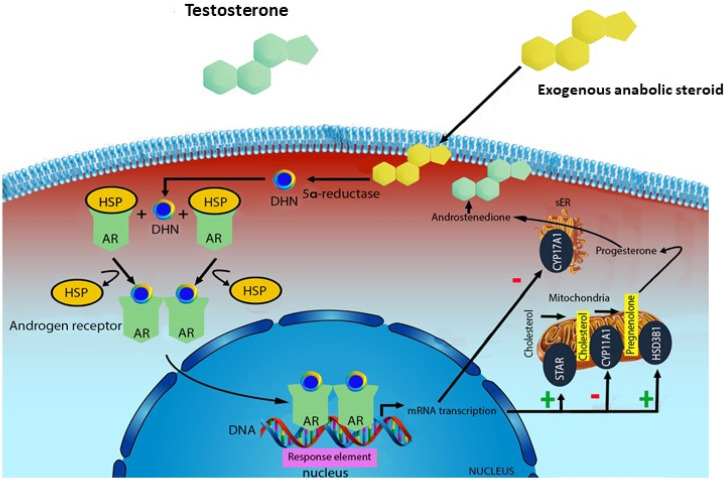
Mechanism of action of exogenous anabolic steroids: an anabolic steroid is transported into the target tissue cell cytoplasm where it can either bind the androgen receptor, or be reduced by the cytoplasmic enzyme 5-alpha reductase. The N-receptor complex undergoes a structural change that allows its translocation into the cell nucleus, where it directly binds to specific nucleotide sequences of the chromosomal DNA. The produced DNA interferes with the physiological biosynthesis of testosterone.

The most relevant mechanisms that lead to the increase of AASs in circulation are: administration of testosterone or its synthetic derivatives or administration of drugs that raise endogenous testosterone production [11]. The mechanism of action of AASs in supraphysiological doses is characterized by the impairment of testosterone biosynthesis in tissues (Figure 1).

AASs exert their effects by activating androgen receptor (AR) signaling. Several parts of the body are involved because of the presence of ARs in many tissues [12]. At normal physiologic levels of testosterone androgen receptors are saturated and the AASs effects may be a consequence of other mechanisms rather than androgen receptors activation. High testosterone levels may have an antagonist effect on glucocorticoid receptors, leading to inhibition of glucose synthesis and protein catabolism. Indeed, high dose AASs may displace glucocorticoids from their receptors, decrease proteins breakdown in muscles, leading to an increase in muscle mass and muscle strength [18]. The inhibition of glucocorticoid action is also due to the stimulation of growth hormone (GH) and insulin-like growth factor (IGF)-1 axis. In this regard, AASs induce an androgen-mediated stimulation of GH and the hepatic synthesis of IGF-1, leading to muscle proteins formation and anabolic effects [5]. Moreover, testosterone is converted by aromatase action to estradiol and estrone, influencing brain and sexual differentiation, bone and muscle mass increase, puberty and sexual functions. High doses of AASs exert an antiestrogenic effect due to a down-regulation of androgen receptors and a competition with estrogens with their receptors [18]. 

Thereby, AASs effects are the result of the amplification of testosterone and estrogens physiologic consequences. Several experimental human studies showed the influence of testosterone and AASs doses concentration on their effects. According to a double-blind human study, low dose administration of methyltestosterone is considered 40 mg/d and high dose is 240 mg/d [19]. In this study, 3 days’ administration of high doses of methyltestosterone led to neuropsychiatric effects. Another study found psychological effects after 14 weeks of 500 mg administration of testosterone cypionate for week [18]. Moreover, a recent study found that in a population of AASs users the weekly dose assumption ranged 75–1550 mg/week [20].

Several studies have suggested an influence of AASs on oxidative stress [18,19,20,21,22]. AASs may alter the function of mitochondrial respiratory chain complexes and mono-oxygenase systems, thereby causing an imbalance between free radical production and their subsequent amelioration [18]. A recent study conducted on Wistar rats treated with ND demonstrated a disruption of the redox metabolism in the animals’ tissues through the increase of reactive oxygen species (ROS) that may play a role in DNA damage [19]. In this regard, the beneficial effect of physical activity in diminishing oxidative stress as a consequence of the upregulation of antioxidant enzymes is well known. However, a role of ND in physical activity-induced cardio-protection impairment has been demonstrated, therefore AASs may play a role in cardiac ischemic injury mediated by oxidative stress [21]. Such data have also been confirmed in a human study that investigated the effects of a supraphysiological administration of testosterone enanthate (500 mg) in healthy volunteers. In this study an impairment of endothelial function as a consequence of the dysfunction of antioxidative capacity following testosterone administration was demonstrated [22]. Furthermore, oxidative stress plays a leading role in AAS-mediated neurotoxicity: androgens may be neuroprotective in cases of low levels of oxidative stress, however, they may increase brain damage in cases of elevated oxidative stress [23].

AAS-related damage is also associated with apoptosis activation [23,24,25,26]. Indeed, it was demonstrated that supraphysiological concentrations of AASs may induce neurotoxicity by involving the apoptotic process and neurodegeneration [24]. Moreover, AASs are responsible for excitotoxicity induced by N-methyl-d-aspartate (NMDA) in primary cultures of mouse cortical cells [24]. A recent study suggested how AASs may induce apoptosis and oxidative stress in the rat brain because of the activation of the non-genomic pathway and the elevation of the intracellular calcium concentration [25]. Another study confirmed a role of apoptosis in AAS-related damage. Androgens may increase the expression of profibrotic cytokines such as TGF-b in mice kidneys, leading to the activation of the apoptotic process and the promotion of focal segmental glomerulosclerosis. Moreover, environmental and inflammation stress can lead to proteotoxic damage and dysregulate heat shock proteins [26]. In this regard, an activation of the extrinsic pathway of the apoptosis in the vascular smooth muscle cells in rats treated with testosterone was observed [27]. 

AASs are characterized by the activation of protein synthesis. The supraphysiological administration of ND decreased the fat mass and increased the protein mass in treated Wistar rats due to amino acid uptake and protein synthesis amelioration [20]. A similar mechanism was described in skeletal muscles: increased protein synthesis, a decrease in protein breakdown, the elevated formation of new myotubes and myonuclei lead to an increase in muscle mass and strength as well as an increased exercise capacity [21]. Moreover, testosterone has anti-inflammatory effects and improves insulin sensitivity because of its capacity to reduce the expression of proinflammatory cytokines, such as interleukin-1β, interleukin-6, and reduce the circulation of inflammatory cells [21].

**Figure 2:**
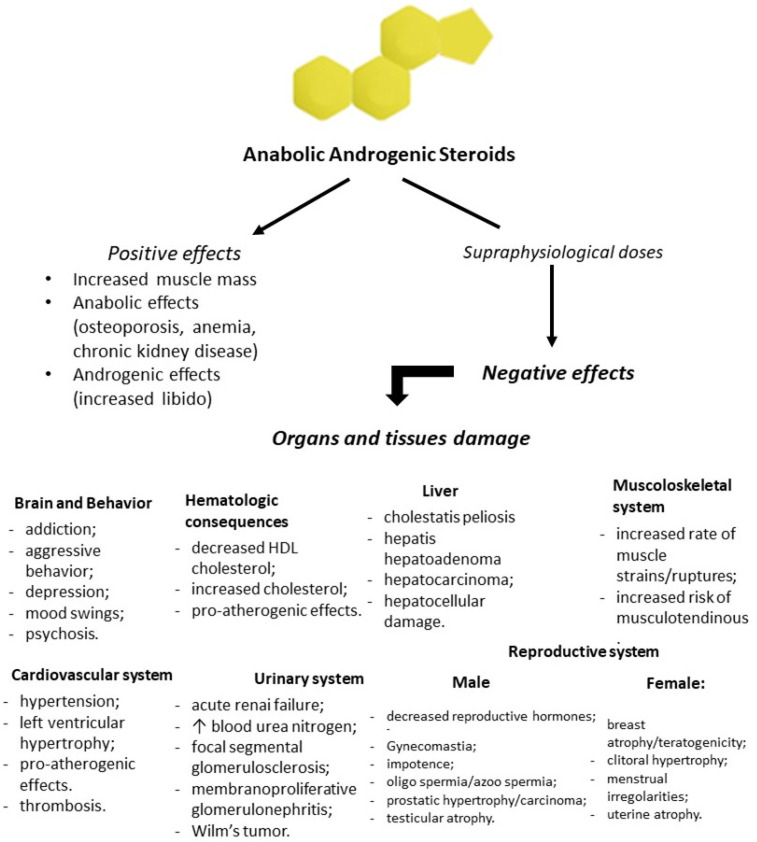
Flowchart of positive and negative effects of anabolic-androgenic steroid (AAS) administration. Prolonged and high doses of testosterone and his derivatives lead to serious consequences in all body tissues and organs.

Recent clinical and experimental studies proved that the increased activity of the renin-angiotensin-aldosterone system (RAAS) plays a pivotal role in the pathogenesis of cardiological diseases. The over-activation of the RAAS may lead to changes in the cardiovascular system observed in subjects taking AASs for doping purposes. The pathogenesis of cardiological changes and many tragic cases resulting from AAS abuse could depend on the strong stimulation of the RAAS and enforced effects by the tissue aldosterone action. A raised level of aldosterone is considered to be related to the occurrence of cardiac illnesses, independently of increased blood pressure. Lastly, the results of a study demonstrated that the presence of AAS metabolites in urine may be a predictive factor of cardiac changes in AAS abusers [27]. Oxidative stress, apoptosis, inflammation and changes in endocrine homeostasis are responsible for multi-organ damage in AASs abusers. Although several positive effects of AAS use had been described, supraphysiological doses and AASs abuse and misuse may lead to serious consequences in all body tissues and organs (Figure 2). 

## 4. AAS Use and Adverse Effects

Several mechanisms are involved in AAS adverse effects and need to be better clarified. AAS related effects involve several organs and systems, both in animals and humans. This is possibly due to the widespread presence of AR in the body and to the impairment of biosynthesis, transformation and degradation of endogenous steroids [28]. AASs bind to a specific type of androgen receptor and by the time the receptors are saturated, AASs in supraphysiological doses may lead to secondary effects [29,30,31]. However, side effects associated with AAS use (i.e., under medical supervision) have to be differentiated from those caused by abuse (i.e., consumption of many drugs at high doses; any nonmedical use of these substances) [32]. Some athletes consume multiple drugs in addition to anabolic steroids such as alcohol, opioids, cocaine, marijuana, and gamma hydroxybutyrate, some of which can interact adversely with AASs. Polydrug assumption makes it hard to attribute the observed effects to a single drug. AAS effects are also related to sex, dose and duration of administration. In this regard, most of the effects are observed after long-term administration [33].

### 4.1. Autopsy Findings

As we mentioned before the prolonged misuse and abuse of AASs can lead to several adverse effects, some of which may be even fatal especially the ones regarding the cardiovascular system, such as sudden cardiac death and coronary artery disease [34,35]. A recent autopsy series described that cardiovascular disease was widespread in AAS-related deaths [36]. Another series showed that all cases had the same findings: absence of asymmetrical left ventricular hypertrophy, coronary atherosclerosis causing significant luminal narrowing, pulmonary thromboembolism, coronary and endocavitary thrombi, and inflammatory infiltrates. Furthermore, the histopathologic study showed myocardial damage characterized by myocyte hypertrophy, focal myocyte damage with myofibrillar loss, interstitial fibrosis, mostly at the subepicardial, and small vessel disease [37]. Another study reviewed all the 19 AAS-related deaths cases presented in the literature, highlighting that in all cases extracardiac causes were excluded, except for one case regarding venous thromboembolism [35].

It was demonstrated that AASs increase the risk of premature death, especially among subjects with other pathologies and/or psychiatric diseases [36]. A survey conducted in 21 gyms in Britain reported that 8% of respondents declared having taken AASs in their life. Another study in the UK showed that 70% of the clientele in a health and fitness community were AAS users [37].

The toxicological investigation executed mostly on urine samples but also on blood and hair samples, by performing several screening tests and analytical methods, showed the presence of AASs and/or their metabolites in urine specimens in 12 cases; in one case nandrolone was detected in blood, while in another case stanozolol was detected in a hair sample [35]. Another study showed that 35% of the users examined were found to be positive for two or more AASs in connection with autopsy. Moreover, an association between the use of AASs and other illicit drugs, such as cannabis, cocaine, amphetamines or LSD, was observed. The combination of physical activity and prolonged/chronic or previous misuse of AASs leads to a predisposition to different patterns of myocardial injury and sudden cardiac death [35]. When performing an autopsy in a sudden death case involving a young athlete, attention to the physical phenotype such as muscular hypertrophy, striae in pectoral or biceps muscles, gynecomastia, testicular atrophy, and acne is mandatory in order to suggest AAS abuse and perform a detailed examination of the heart. Chemico-toxicological analysis is a crucial tool to assess the link between sudden cardiac death and AAS abuse [38]. Autopsy plays a pivotal role in the study of AAS adverse effects and organ damage related to their use/abuse. Moreover, autopsy studies may provide useful information regarding the pathophysiology of the effects of AAS long-term administration, therefore autopsy practice should be implemented in suspected AASs-related deaths.

### 4.2. Brain and Behavior

The neurotoxic action of AASs is associated with both membrane AR and G-protein coupled receptors [39]. Furthermore, several studies highlighted the role of apoptosis in determining brain damage [24,25,32,40,41]. Indeed, it was demonstrated that high concentrations of methandienone and 17-a-methyltestosterone provoke detrimental effects on neuron cell cultures expressing AR, inhibiting neurite network maintenance, leading to cell death through apoptosis and cleavage of protective chaperone proteins, such as Hsp90 [24]. 

A recent study suggested that miRNA dysregulation may be involved in the mechanisms that characterize AAS-related brain damage. In this study three groups were investigated: “AAS” users, “Cocaine” abusers and “Aging” people. In this regard, miR-34 and miR-132 were considerably higher in the “AAS” group [42].

The presence of apoptosis in brain areas of rats treated with long-term administration of nandrolone was suggested in a recent study. In this regard, a link between oxidative stress and NF-Kb signaling was described, promoting brain injury in specific areas, such as the hippocampus, striatum and frontal cortex [32]. Furthermore, it was found that daily injections of stanozol in male adult rats for 28 days led to histopathologic changes in the hippocampus by activating apoptotic and pre-apoptotic cells [40]. Moreover, another study demonstrated that supraphysiological doses of AASs impair the beneficial effects of physical activity on hippocampal cell proliferation and apoptotic signaling [41]. Endurance exercise improves the redox system balance by stabilizing the mitochondrial membrane, leading to a reduction of apoptotic effects of ND in neural cells [25].

Cognitive function may also be impaired by AAS abuse. Weightlifters exposed to AASs had lower cognitive functions, such as motor and executive performance, compared to nonexposed subjects [43]. According to a recent study that performed a neuroimaging investigation of AAS users, smaller overall gray matter, cortical and putamen volume, and thinner cortex in widespread regions in AAS users compared to non-using weightlifters was observed [44]. Furthermore, another imaging study showed markedly increased right amygdala volumes; markedly decreased right amygdala and reduced dACCgln/glu and scyllo-inositol levels compared to nonusers [45]. Recent evidence, by administrating neuropsychological tests to weightlifters both AAS users and nonusers, demonstrated a cognitive disfunction due to long-term high AAS exposure [46]. In this regard, oxidative stress and apoptosis due to AASs abuse may lead to neurodegeneration and dementia, especially in long-term users, adolescents and young adults [47,48].

AASs in supraphysiological concentrations influence several central nervous system functions, such as memory, aggressiveness, anxiety and depression, particularly in predisposed individuals [48,49,50,51,52]. The underlying mechanisms involve neurotransmission by affecting the synthesis and degradation of neurotransmitters, as well as neurotransmitter metabolism [53]. In addition, an animal study suggested that long-term administration of ND leads to anxiolytic behavior and memory impairment. [54,55]. Chronic administration of high doses of AASs is related to anxiety-like behavior through the corticotrophin release factor by enhancing GABAergic inhibitory effects from the central amygdala onto the bed nucleus of the stria terminalis [56]. Moreover, chronic AAS administration changes neurotransmitter expression involved in aggression control [57,58,59]. Lastly, AASs may induce NMDA receptor phosphorylation in order to increase excitatory neurotransmission, resulting in an increment of aggression [60]. In this regard, the orbitofrontal cortex may play a role in the aggressiveness and violent behavior due to AAS consumption. Indeed, the reduction of the orbitofrontal cortex observed in such cases may lead to the lack of inhibitory control [50]. People who use AASs have a higher probability to be drug and alcohol abusers [4,33,57]. Long-term research is needed to clarify the mechanisms and the organic and social processes involved in neuropsychiatric effects of AAS abuse.

### 4.3. Cardiovascular System

Notwithstanding the elevated morbidity and mortality, cardiac and metabolic consequences of AAS abuse are still unclear [59,60,61,62]. Cardiac injury is the most frequent consequence of the administration of exogenous steroids, due to its susceptibility to oxidative stress and its important metabolic activity, compared with the remaining body tissues and organs [63]. Chronic administration of high doses of AASs is responsible for the dysfunction in tonic cardiac autonomic regulation. Indeed, an experimental study demonstrated that rats treated with AASs were characterized by the impairment of parasympathetic cardiac modulation, decreased high frequency power and heart rate variability [64]. Furthermore, the inflammatory process may play a role in triggering cardiac injury in AAS abusers. In fact, in a mouse model a strong cytokine reaction was observed in mice treated with anabolic steroids compared to the control group, suggesting a role of TNF-α in determining myocardial injury [65]. Moreover, it was demonstrated that after administration of anabolic steroids treated animals lost the adaptive response of exercise-induced amelioration of antioxidant activity [21,32,58]. AAS use in supraphysiological doses is associated with abnormal plasma lipoproteins [59,66,67]. A human study including hypogonadal men undergoing substitutive therapy with testosterone showed decreased plasma levels of high-density lipoprotein (HDL) cholesterol [68]. Other studies found hyperomociysteinaemia and increased low-density lipoprotein (LDL) cholesterol levels after long-term AAS administration, underlining the promotion of atherogenesis of these substances [66,67,68]. An increased sympathetic activity was observed after AAS administration [69]. Moreover, high concentrations of AASs by activating ARs, cell membrane receptors and secondary transmitters stimulate the renin-angiotensin-aldosteron system, leading to an increased synthesis of heart muscle, left ventricular hypertrophy and hypertension [27]. AAS users show higher left ventricular mass index, thicker left ventricular walls, more concentric left geometry and myocardial mechanical dysfunction compared to non-users [70,71,72]. Long-term training associated with AAS administration reduce left ventricle relaxation properties [73]. In this regard, the use of anabolic steroids is associated with the loss of the beneficial effects on left ventricle function induced by exercise [74]. Arrhythmic events following long-term administration of AASs were reported [68,75,76,77,78]. Atrial fibrillation is the most frequent event but ventricular arrhythmias and sudden cardiac death were described in literature. Both human and animal studies showed an association between testosterone administration and the impairment of cardiac repolarization [76,77,78,79]. Moreover, cardiac hypertrophy induced by AASs plays an important role in electric and morphologic heart disturbances [77]. AAS users are characterized by an increased volume of atherosclerotic plaque [80]. In addition, 3% of AAS users had myocardial infarction as a consequence of atherosclerotic disease [81]. The mechanisms involved in AAS-induced myocardial infarction are the following: atherogenesis, thrombosis and vasospasm. Experimental data showed that in animal treated with AASs there were increased thrombotic stimuli. Furthermore, AAS abuse is related to endothelia dysfunction by impairing both endothelial-dependent and endothelial-independent vasodilatation [62]. AAS abuse raises the risk of life-threatening arrhythmias and sudden cardiac death. The hypothesized mechanisms are pro-arrhythmic effects of AASs, induction of myocardial ischemia, structural changes and repolarization impairment [78,79].

A recent study suggested that Doppler myocardial imaging is a useful tool to detect subclinical left ventricular dysfunction in AAS athlete abusers [79]. New imaging tools, such as magnetic resonance, may give fundamental information regarding myocardial tissue in these cases [76]. Clinicians must be aware of the mechanisms involved in cardiotoxicity, the pathological and clinical consequences, as well as the diagnostic tools to highlight cardiac damage in AAS abusers, in order to consider AAS abuse in differential diagnosis and undertake primary and secondary prevention in their patients.

### 4.4. Liver

Hepatotoxicity is one of the most frequent side effects of AAS abuse [82,83]. AAS-induced hepatotoxicity was hypothesized to be related to oxidative stress in hepatic cells. Following AR receptor activation an increase in reactive oxygen species can be observed due to the increase in mitochondrial b-oxidation. Moreover, antioxidant substances have a protective role against hepatotoxicity mediated by AASs. It was also demonstrated that androgenic potency and metabolic resistance are positively linked to the degree of liver damage [82]. 

AAS-induced hepatotoxicity is influenced by genetic factors, and is related to the infiltration of inflammatory cells in liver tissue, such as lymphocytes, neutrophils and eosinophils [83,84]. Oxidative stress could have a role in determining liver damage consequent to AAS abuse by activating androgen receptors that lead to mitochondrial degeneration of hepatic cells [84]. A recent study evaluated the liver effects of 5 weeks of administration of ND in rats. The results highlighted an increase of plasma levels of liver necrosis markers, an increase in collagen deposition in liver parenchyma, portal space, and the centrolobular vein [84]. The mechanism involved in collagen deposition could be the increase in the number and in the activity of Kuppfer cells. In this regard, Kuppfer cell activation leads to the production of many inflammatory cytokines such as TGF-b1, NF-Kb, IL-1b, related to the liver fibrosis process [85,86].

Two of the most common liver consequences following supraphysiological doses of AASs are peliosis and cholestasis. Peliosis is characterized by multiple blood-filled cavities histologically characterized by the presence of scattered, small, blood-filled cystic spaces throughout the liver parenchyma [83]. The mechanism involved could be the induction of hyperplasia of the hepatocytes responsible for mechanical obstruction of hepatic veins and the genesis of nodules and tumors [83,84,85]. In addition, animal studies demonstrated that bile accumulation can be a consequence of the reduction of his transportation ability. However, AAS-associated cholestasis is not characterized by the presence of necrosis and inflammation. Inflammation and necrosis may lead to a regenerative signal in AAS-induced hepatotoxicity [83,87]. A correlation between hepatocellular adenoma and androgen steroid therapy was described in the literature and the risk of androgen-associated liver tumor seems to be related to the dose and the potency of AAS administration [83,84]. Further studies are warranted to clarify the correlation between AASs administration and hepatocellular proliferation, in order to undertake preventive measures.

### 4.5. Urinary System

Several studies highlighted that prolonged androgen exposure has a direct toxic effect on kidneys, especially glomerular cells, causing accumulation of mesangial matrix, podocyte depletion and structural adaptations [26,87,88,89]. In this regard, kidney tissues are characterized by the expression of ARs. AR activation leads to cell growth and hypertrophy in the kidney. A recent report suggested that ND exposure promotes hypertrophy in proximal and distal convoluted tubules of mice kidneys [90]. Moreover, both testosterone activity and direct ND action to AR may play a role in the genesis of kidney fibrosis after long-term ND exposure [89].

Prolonged administration of ND in mice has been shown to cause dose-dependent oxidative kidney stress and damage. Mice kidneys treated with ND exhibited increased lipid peroxidation and decrease antioxidant enzyme activity, such as glutathione reductase and glutathione peroxidase [87]. A recent study suggested a dose related oxidative stress in mouse kidneys treated with prolonged doses of ND [87]. The authors observed an increase in lipid peroxidation markers and an increase of pro-inflammatory and pro-apoptotic markers such as IL-1B, Hsp90 and TNF associated with a decrease of antioxidant enzymes, which could lead to secondary focal segmental glomeruloscelerosis [87].

Morphological changes were observed in mice treated with ND. Three months after intramuscular injection of androgen, several histopathological alterations were detected: glomerular atrophy and fragmentation, tubular wall rupture, vacuolar degeneration of the epithelium lining of the proximal convoluted tubules and blood hemorrhage between the tubules, basal lamina thickening in distal convoluted tubules and tubes with only the basal lamina, many hyaline cylinders, some areas of necrosis, eosinophilic cell cytoplasm, which is a sign of chronicity and vascular congestion, were found in kidney samples [91]. As in other tissues and organs, oxidative stress, apoptosis and inflammation play a pivotal role in urinary system damage. This information is fundamental for therapeutic and prevention measures.

### 4.6. Muscoloskeletal System

Muscle mass seems to be influenced by AAS administration [30,92,93]. In fact, testosterone, by binding to AR, produces an increased production of IGF-1, a decreased expression of myostatin and the differentiation of pluripotent mesenchymal cells into a myogenic lineage. These mechanisms are involved in an increase in protein synthesis, a decrease in protein breakdown, the formation of new myotubes as well as the increase in myonuclei number, thereby leading to the increase in muscle mass, strength and exercise capacity [94].

In addition, high concentrations of AASs can provoke serious skeletal muscles injuries [95]. An experimental study demonstrated that supraphysiological doses of AASs induce a decrease in MMP-2 activity in the agonist jumping rat muscles [96]. It was suggested that the vascular endothelial growth factor (VEGF) may play a role in the mechanism involved in skeletal exercise adaptation. VEGF expression was reduced in rats who underwent ND administration and this is possibly related to MMP-2 activity dysfunction, since MMPs are involved in the regulation of VEGF extracellular stores [97]. Moreover, the decreased expression of VEGF may play a role in skeletal damage due to AASs, as a consequence of poor remodeling and poor vascularization [97]. Nevertheless, AASs could also be involved in tendon damage [98,99]. The morphology and the organization of collagen fibers may be modified by physical activity. In this regard, AAS abuse also increases the risk of tendon rupture, due to the increase of muscle mass, strength and the inability to respond, especially during exercise [98]. It was demonstrated that ND increased tendon remodeling despite decreases in MMP-2 activity in rat tendons [99]. However, AAS-related MMP dysregulation still needs to be better clarified. Esthetic purposes, increase of muscle mass and strength are one of the most frequent reason why young people and athletes are AASs abusers. Information campaign and public health measures are needed to increase the awareness in young population regarding muscoloskeletal side effects of AASs abuse.

### 4.7. Reproductive System

Androgens play a pivotal role in the development of male reproductive organs. They are necessary for puberty and male sexual function. AAS administration leads to a negative feedback on the hypothalamic-pituitary axis, altering the secretion of both FSH and LH, causing infertility [5]. A recent study focused on Leydig cell cultures treated with ND, demonstrating an impairment of testosterone production due to STARR and CYP17A1 expression interference in these cells [100].

Previous studies suggested that both current and past AAS users reported increased frequency of morning erections, sexual thoughts, and satisfaction. However, several side effects were observed such as erectile dysfunction, anorgasmia and premature ejaculation. Nevertheless, recent findings support the hypothesis that increased frequency and duration of high-dose AASs lead to sexual dysfunctions following discontinuation [101]. Prior AAS use is frequent among young men with hypogonadism and this element needs to be taken into consideration in the clinical evaluation of hypogonadism [102].

Animal histological studies of testes demonstrated spermatogenesis impairment with lack of advanced spermatidis and decreased number of spermatidis due to AAS use [5,88]. An impairment of the blood-testis-barrier was also observed in CD1 mice treated with ND, which may play a role in triggering the spermatogenesis alteration [103]. Moreover, quantitative changes in number, diameter and thickness of seminiferous tubules were detected in albino rats after AAS administration [104]. Apoptosis has been reported to play an important role in the regulation of germ cell populations in the adult testes. The correlation between apoptosis and high AAS doses and exercises has recently been experimentally assessed in animal models. Shokri et al. report a significant increase in the rate of apoptosis of spermatogenic cells after nandrolone administration, an increase clearly amplified by physical exercise [5].

According to the length of use of anabolic steroids and the period since the last drug administration prior to the survey, changes in sperm parameters were observed in a study: the percentages of motile sperm decreased among bodybuilders compared to healthy volunteers. Moreover, in relation to these results even after prolonged use of extremely high concentrations of anabolic steroids, sperm production can return to physiological rates for bodybuilders who stop consumption of anabolic steroids for 4 months [5]. Furthermore, some authors found alterations in sperm quantity, protamine, and DNA integrity in Wistar rats that underwent exercise treated with high concentrations of ND. The incidence of AASs in protamine and DNA fragmentation is a relevant issue in the study of male fertility, given that these drugs are used in high doses as in the case of some athletes. In addition, a direct relationship between irregular protamine expression and sperm count, motility, morphology, or fertilization was reported [104].

According to recent data, 20% of patients who were being treated for symptomatic hypogonadism had previously used AASs. Recognition of the specific details of the user’s AAS exposure is crucial for their medical management. Management strategies for male infertility secondary to AAS induced hypogonadism should focus on the underlying hypogonadal state [105,106]. According to a recent study, chronic AAS abuse should be considered when a muscular man presents with hypogonadism, onset of gynecomastia or hirsutism.

### 4.8. Hematologic Consequences

Before the introduction of recombinant human erythropoietin, AASs were used in the treatment of anemias, indeed, AASs are capable of increasing erythropoietin secretion. Other AAS induced side effects are the increase of hematocrit and erythrocytosis [93]. AAS abuse has been recurrently associated with an increased risk of thrombosis and is detrimental to cardiovascular health [107,108,109]. However, the association has primarily been based on case reports. Increased LDL and decreased HDL are linked to an increased cardiovascular risk. Mild, but significant, increases in mean red blood cell, hematocrit, hemoglobin, and white blood cell concentrations in 33 men were described after intramuscular testosterone enanthate, 200 mg every 3 or 4 weeks for 24 weeks [93]. The influence of AASs on plasma concentration and function of coagulation factors depends on the substance and the dose of the anabolic steroid [110]. In this regard, it was demonstrated that physiological testosterone stimulates tissue plasminogen activator and tissue factor pathway inhibitor and inhibits plasminogen activator inhibitor type 1 release in endothelial cells. The relationship between AAS abuse and thrombosis has not been sufficiently clarified by the current literature of which only a few reports concern actual thrombotic outcomes [11]. A recent report suggested a possible correlation between AAS abuse and immunodeficiency that may be related to a mimicking action of corticosteroid activity. Moreover, this report suggested that AAS abuse should be investigated when an uncommon death occurs in immunosuppressed patients [111,112].

### 4.9. AASs and Cancer

The biochemical mechanism of AASs is similar to that of testosterone. AASs bind to DNA sequences and induce gene expression alterations. In a recent review regarding androgen effects on cellular functions, it was stated that a combination among genetic and epigenetic factors is the cause of toxicity, mutagenicity, genotoxicity and carcinogenicity of sexual hormones [113]. However, AAS related genotoxicity still remains unclear. Epigenetic molecular mechanisms, which lead to a genetic transcription control are: DNA methylation, histone modifications and chromatin condensation [114]. DNA methylation inhibits the binding between transcriptional factors and their target sequences, both promoters and introns, preventing transcriptional expression activation. Chromatin condensation also regulates transcriptional expression [115,116]. Testosterone synthetic derivatives can be metabolized, in adipose, cerebral and testicular tissues, to 17β-estradiol, a known potentially mutagenic and carcinogenic steroid [113]. 17 beta-estradiol and its metabolites are also considered inducers of cell proliferation. Furthermore, during their catabolism, AASs reveal their oxidative role, increase reactive oxygen species (ROS) production, which are highly unstable, easily lose hydrogen atoms, form covalent bonds with DNA bases or sequences, and may induce genetic damage [113].

It has been suggested that the incidence of cancer in different tissues is strictly positively correlated to the number of stem cell divisions in the lifetime occurring in them [50]. On this basis, it can be hypothesized that the chronic administration of nandrolone, favoring the persistence and viability of stem cells in different tissues, could represent a preconditioning that, in addition to multiple hits, could enhance the risk of carcinogenesis onset especially in stem cell-rich tissues such as the liver [117,118]. The side effects on the natural synthesis of anabolic steroids play a potential role in hormonal changes/regulation and it could be suspected to be at the base of certain carcinogenic mechanisms [113,119]. Furthermore, easily accessible and commonly diffused AASs, such as nandrolone and stanozolol, have the potential role in the pathogenesis of cancer, such as Leydig cell tumor through multiple process pathways [113]. Given that it was demonstrated a correlation between AASs abuse and cancer, the prevention of its abuse and the information campaigns in gyms and among young athletes are mandatory. In this regard, surveillance of long-term abuser is warranted in order to perform at early diagnosis. 

Adverse effects of anabolic steroid use are summarized in Table 1.

## 5. Conclusions

This review suggests that AAS misuse and abuse lead to adverse effects in all body tissues and organs. Oxidative stress, apoptosis, and protein synthesis alteration are common mechanisms involved in AAS-related damage in the whole body. This review shows that long-term administration of high doses of AASs may lead to serious consequences, such as hypogonadism, cardiac impairment, neurodegeneration, coronary artery disease and sudden cardiac death. The most reported long-term side effects affect the cardiovascular system, such as cardiomyopathy and atherosclerotic disease. Hypogonadism is a frequent finding in AAS abusers and need to be taken into consideration when AAS use is suspected in order to undertake aggressive treatment [8,120].

Several experimental studies focused on the mechanisms involved in neuropsychiatric effects of AASs. The pathways and the molecular processes are still unclear and need to be clarified [121,122,123,124]. In this regard, further studies are needed to assess the epidemiology of antisocial behavior related to AAS assumption and the relationship with other drug consumption. 

Moreover, considering that most of the customers are young sportsman and that most of these drugs are easily obtained online, AAS abuse is a considerable public health issue [3]. 

Clinicians and family doctors should be aware of AAS adverse effects, in order to investigate AAS use in high risk patients, especially in young athletes [121]. In this regard, cardiac imaging may be a helpful tool to assess the presence of subclinical morphological cardiac alterations in AAS abusers. In addition, recent studies reported that miRNAs may play a role in multiple human diseases including AAS adverse effects, suggesting a possible role of these markers in identifying serum or tissue biomarkers with anti-doping potential. However, further studies are needed in this field, given that there is no reliable test to diagnose AAS abuse. 

Given the high mortality of atherosclerotic disease and AAS-induced cardiomyopathy, as well as the risk of sudden cardiac death reported in the literature, primary and secondary prevention are crucial in AAS users in order to avoid serious consequences. The scientific community should intensify its efforts to assess the pathophysiology of behavior and cognitive impairment due to long term AAS exposure. Moreover, evidence is urgently required to support the development of a reliable diagnostic tool to identify precociously AAS abuse as well as evidence-based therapy [57,125,126,127,128,129,130,131]. 

Information and education are fundamental tools for AAS misuse preventions. As long as anabolic steroid misuse is popular among young athletes, information campaigns regarding AASs and other doping agents should be encouraged in high schools. In this regard, to prevent the use of AASs public health measures in all settings are crucial. These measures consist of improved knowledge among healthcare workers, proper doping screening tests, educational interventions, and updated legislation.

Although the use of AASs appears to increase the risk of premature death in various categories of patients, further research about this problem is urgently needed [132,133,134,135,136,137,138,139]. 

## Figures and Tables

**Table 1:** Summary of adverse effects of anabolic steroid use.

Organ/System	Author(s)	Year of Publication	Adverse Effects
Brain and Behavior	Bertozzi, et al.	2019	↓ orbitofrontal cortex; lack of inhibitory control.
	Hauger, et al.	2019	↓ memory, ↓ anxiety ↑ depression.
	Joksimovic, et al.	2019	↓ orbitofrontal cortex; lack of inhibitory control.
	Karimooy, et al.	2019	Neurodegeneration; histopathologic changes in hippocampus.
	Bertozzi, et al.	2018	↑ aggressiveness.
	Bjørnebekk, et al.	2017	Lower cognitive functions, motor and executive performance; ↓ gray matter, cortical and putamen volume.
	Bueno, et al.	2017	Neurodegeneration.
	Turillazzi, et al.	2016	Neurodegeneration; hippocampus, striatum and frontal cortex injury.
	Joukar, et al.	2017	Neurodegeneration.
	Kaufman, et al.	2015	↑↑ right amygdala volume.
	Piacentino, et al.	2015	↓ memory, ↑ aggressiveness, ↓ anxiety ↑ depression.
	Gomes, et al.	2014	Neurodegeneration.
	Basile, et al.	2013	Neurodegeneration.
	Kanayama, et al.	2013	Cognitive dysfunction.
	Elfverson, et al.	2011	↑ aggressiveness.
	Melloni, Jr., et al.	2010	Anxiety-like behavior.
	Kouvelas, et al.	2008	↓ memory, ↓ anxiety.
	Sato, et al.	2008	↑ aggressiveness.
	Rashid, et al.	2007	↓ memory, ↑ aggressiveness, ↓ anxiety ↑ depression.
	Henderson, et al.	2006	behavioral effects.
Cardiovascular system	Marocolo, et al.	2018	Arrhythmic events; cardiac hypertrophy.
	Rasmussen, et al.	2018	Hypertension; left ventricular hypertrophy.
	Baggish, et al.	2017	↑ left ventricular mass index; ↑ left ventricular walls; myocardial mechanical dysfunction. myocardial infarction.
	Seara, et al.	2017	↑ left ventricular mass index; ↑ left ventricular walls; myocardial mechanical dysfunction.
	Christou, et al.	2016	Abnormal plasma lipoproteins.
	Esperón, et al.	2016	Atherosclerotic plaque.
	Vasilaki, et al.	2016	Cardiac injury
	Akçakoyun et al.	2014	Arrhythmic events.
	Angell, et at.	2012	Arrhythmic events; impairment of cardiac repolarization.
	Golestani, et al.	2012	↓ plasma levels of HDL cholesterol; ↑ LDL cholesterol levels; arrhythmic events.
	Chrostowski, et al.	2011	Increased synthesis of heart muscle, left ventricular hypertrophy and hypertension.
	Riezzo, et al.	2011	Hypertension; left ventricular hypertrophy; pro-atherogenic effects; thrombosis.
	Achar, et al.	2010	↓ plasma levels of HDL cholesterol; ↑ LDL cholesterol levels.
	Alves, et al.	2010	Increased sympathetic activity.
	D’Andrea, et al.	2007	Left ventricular dysfunction.
	Phillis, et al.	2007	Arrhythmic events.
	Chaves, et al.	2006	Oxidative stress.
	Nottin, et al.	2006	↓ left ventricle relaxation properties.
	Pereira-Junior, et al.	2006	Impairment of parasympathetic cardiac modulation; heart rate variability.
Liver	Solimini, et al.	2017	Mitochondrial degeneration of hepatic cells.
	Bond, et al.	2016	Hepatotoxicity.
	Neri, et al.	2011	Hepatotoxicity.
	Schwingel, et al.	2011	Liver fibrosis process.
	Vieira, et al.	2008	Liver fibrosis process.
Urinary system	Brasil, et al.	2015	Kidney fibrosis.
	Riezzo, et al.	2014	Oxidative stress; Accumulation of mesangial matrix.
	D’Errico, et al.	2011	Accumulation of mesangial matrix.
Muscoloskeletal system	Carson, et al.	2015	↑ Muscle mass.
	Marqueti, et al.	2011	Tendon damage.
	Paschoal, et al.	2009	Skeletal muscles injuries.
Reproductive system	Armstrong, et al.	2018	Sexual dysfunctions.
	Barone, et al.	2017	Spermatogenesis alteration.
	El Osta, et al.	2016	Infertility; ↓ number of spermatidis.
	García-Manso, et al.	2016	Changes in number, diameter and thickness of seminiferous tubules.
	Pomara, et al.	2015	Impairment of testosterone production.
	Rahnema, et al.	2014	Hypogonadism.
	Coward, et al.	2013	Hypogonadism.
Hematologic consequences	Chang, et al.	2018	↑ Thrombosis.
	Casavant, et al.	2007	↑ hematocrit, ↑ erythrocytosis.

Abbreviations: ↑ increase; ↓ decrease.

## Data Availability

Data sharing not applicable, no new data were created or analyzed in this study.
